# Systemic Lupus Erythematosus as a Risk Factor for Encapsulating Peritoneal Sclerosis: A Case Report

**DOI:** 10.7759/cureus.82699

**Published:** 2025-04-21

**Authors:** Aabid Mohiuddin, Fawaz Hussain, Leslie Lin, Ahmed Alhaj, Yahya Osman-Malik

**Affiliations:** 1 Department of Internal Medicine, Detroit Medical Center/Wayne State University, Detroit, USA; 2 Division of Nephrology, Detroit Medical Center/Wayne State University, Detroit, USA

**Keywords:** encapsulating peritoneal sclerosis, fibrosis, peritoneal dialysis, surgical enterolysis, systemic lupus erythematosus

## Abstract

Encapsulating peritoneal sclerosis (EPS) refers to the insidious formation of a thick fibrous cocoon encasing the intra-abdominal organs and may lead to bowel obstruction. EPS is a known but rare complication of chronic peritoneal dialysis (PD) due to the long-term exposure of glucose degradation products and recurrent peritonitis, promoting inflammatory fibrosis and sclerosis of intra-abdominal structures. However, PD patients with systemic lupus erythematosus (SLE) may be at increased risk of developing EPS due to the concurrent immune-mediated inflammation of the peritoneal serosa. This report describes the case of a young female with SLE and end-stage renal disease (ESRD) due to lupus nephritis who developed EPS after five years of PD. Her clinical course was complicated by septic shock and pneumoperitoneum; exploratory laparotomy revealed diffuse fibrous encapsulation of the liver, transverse colon, and stomach requiring surgical enterolysis. This case highlights the role of SLE as an important risk factor for EPS and underscores the need for preventive measures among this high-risk population of chronic PD patients.

## Introduction

Encapsulating peritoneal sclerosis (EPS) is a rare condition characterized by the formation of inflammatory fibrocollagenous layers around the small intestine and other intra-abdominal organs. It typically develops as a complication of chronic peritoneal dialysis (PD), initially presenting with non-specific abdominal symptoms before progressing to entrapment of the abdominal contents and subsequent ileus and obstruction [1]. Interestingly, patients with systemic lupus erythematosus (SLE) who develop end-stage renal disease (ESRD) due to lupus nephritis appear to experience a higher incidence of EPS while on PD. We present a case of a young female with SLE on chronic PD who developed severe complications from EPS.

## Case presentation

A 32-year-old female with a medical history of SLE and comorbid ESRD secondary to lupus nephritis was admitted to the medicine service with severe, diffuse abdominal pain, cramping, nausea, and non-bloody, non-bilious emesis. She had been on PD for the last five years. Three days prior, she had been discharged from an outside hospital with a diagnosis of culture-negative peritonitis after presenting with similar symptoms. She had been prescribed empiric oral and intraperitoneal antibiotics to continue at home, but had not taken them due to a misinterpretation of discharge instructions.

On admission, she was afebrile and hemodynamically stable. Physical examination revealed a woman in mild distress due to discomfort, but alert, oriented, and able to answer questions appropriately. Her abdomen was diffusely tender to palpation with voluntary guarding and no rebound tenderness. Laboratory evaluation was notable for marked leukocytosis with a white blood cell count of 31,000 cells/μL and a neutrophilic predominance. She had a high anion-gap metabolic acidosis, which improved after intravenous fluid resuscitation. Other laboratory abnormalities, such as elevated creatinine and mild hyperphosphatemia, were consistent with her ESRD (Table 1).

**Table 1:** The patient's laboratory evaluation on admission

Parameters	Patient values	Reference range
Metabolic panel		
Sodium, mmol/L	136	136-145
Potassium, mmol/L	3.6	3.6-5.1
Chloride, mmol/L	91	98-107
Anion gap, mmol/L	20	4-12
Bicarbonate, mmol/L	25	22-29
Glucose, mg/dL	96	74-109
Creatinine, mg/dL	9.6	0.5-1.2
Calcium, mg/dL	10	8.6-10.2
Magnesium, mg/dL	2.1	1.6-2.4
Phosphate, mg/dL	5.3	2.4-5.1
Complete blood count		
White blood cell count, units/uL	30.6	1000
Absolute neutrophil count, units/uL	25	1.6-7.1
Absolute neutrophil percentage	80	38-76
Hemoglobin, g/dL	11	11.5-15.1
Mean corpuscular volume, fL	80	81-98

A peritoneal fluid culture was collected on admission before resuming the empiric coverage regimen of fluconazole, vancomycin, and ceftazidime, which had been prescribed at her previous discharge. Her PD was resumed with five daily exchanges using a standard 2.5% dextrose solution. Her abdominal pain was attributed to the previously diagnosed culture-negative peritonitis and subjective constipation, for which an aggressive bowel regimen was prescribed. No abdominal imaging was obtained on admission.

**Figure 1:**
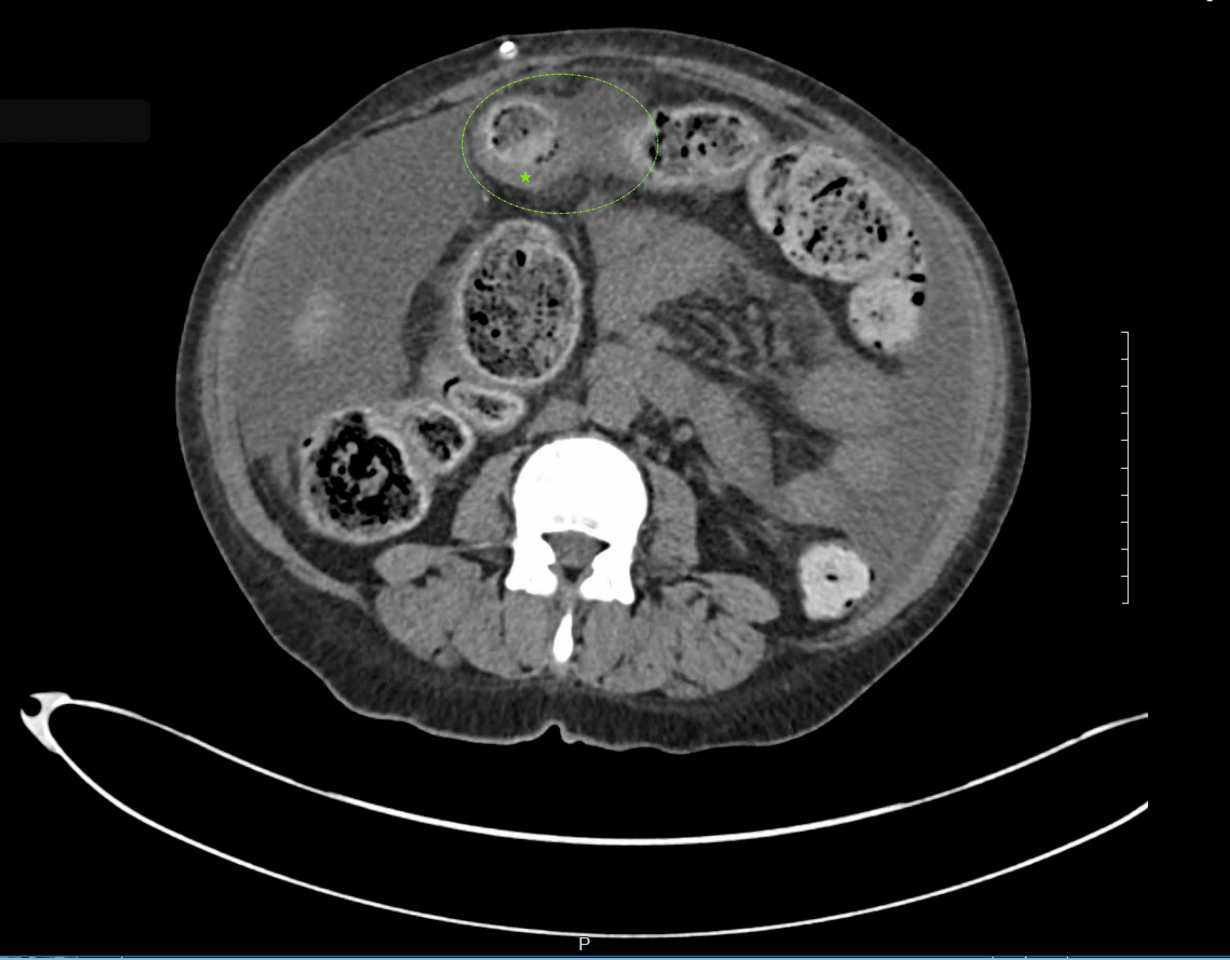
CT imaging of the abdomen revealed several findings including constipation, which can be seen within the dashed green circle. The green star indicates an area of bowel wall thickening CT: computed tomography

On day two, the patient’s leukocytosis initially improved with antibiotics. However, it rebounded on day three, despite her remaining afebrile. Meanwhile, her abdominal pain worsened, and she remained constipated, prompting a non-contrast CT scan of her abdomen. The scan revealed minimal ascites secondary to PD, constipation, and an incidental finding of diffuse large bowel wall thickening ‘likely related to ascites and/or peritoneal dialysis’ (Figure 1). An esophagogastroduodenoscopy was conducted, but it only showed esophagitis secondary to repeated emesis.

**Figure 2:**
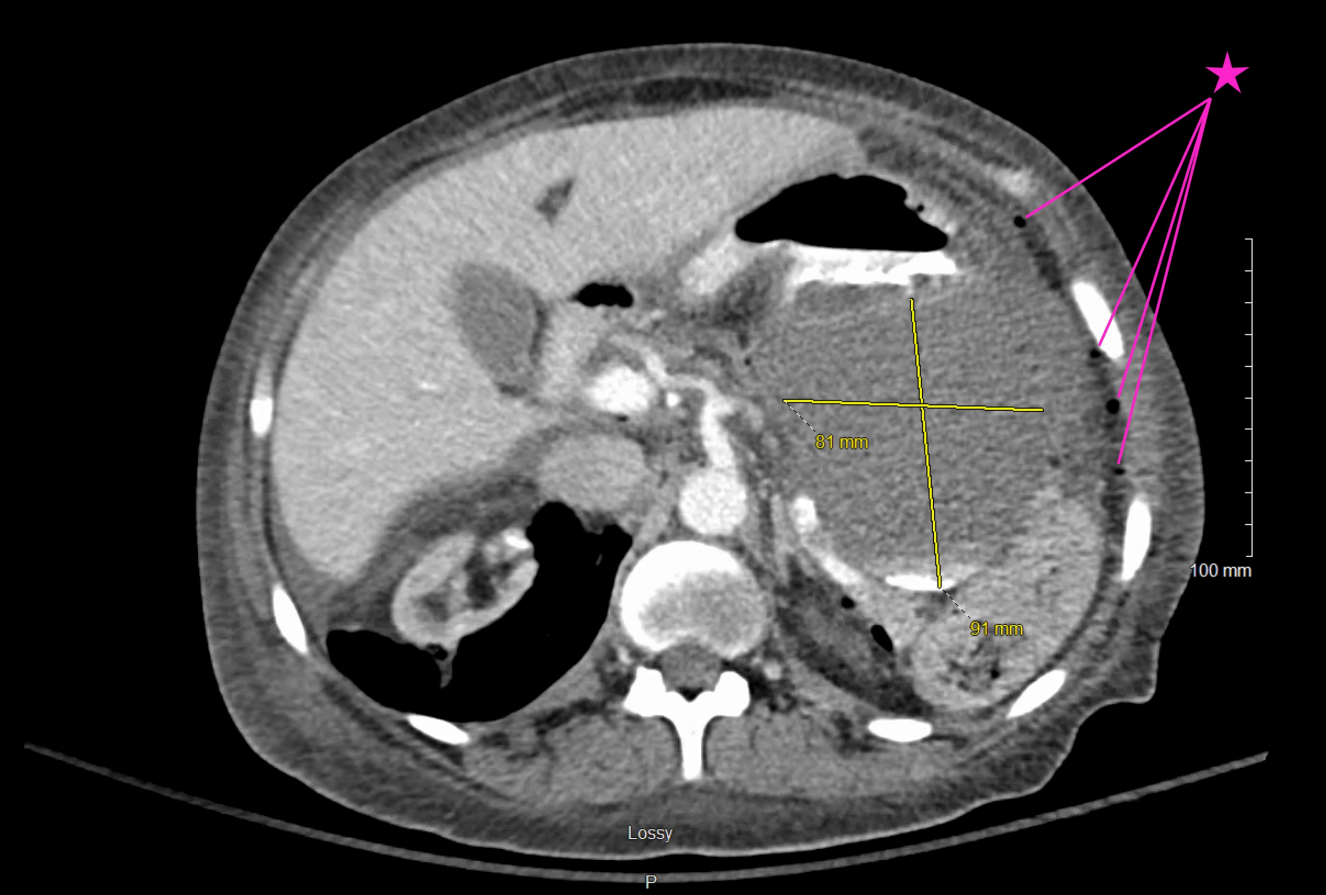
CT imaging reveals the sequelae of a bowel perforation including adjacent fluid collection (indicated by yellow measured cross) with dependent contrast pooling. The pink star points to several small pockets of free air CT: computed tomography

On day four, the peritoneal fluid culture collected on admission grew E. faecalis, and vancomycin was replaced with ampicillin. Due to her persistent abdominal pain and positive culture results, the PD catheter was removed, and dialysis was performed through an existing, functioning arteriovenous fistula. Despite these measures, the patient clinically deteriorated and was transferred to the ICU for the management of septic shock requiring vasopressors. In the ICU, abdominal X-rays showed a severe stool burden, but she did not respond to the escalation of her bowel regimen. Repeat abdominal CT imaging revealed a critical finding of pneumoperitoneum and profound gastroduodenal, jejunal, and cecal mucosal edema due to peritonitis and perforation (Figure 2).

During an emergent exploratory laparotomy, surgeons repaired a 4-cm hole in the transverse colon, which was complicated by an hour spent lysing adhesions caused by a ‘very thickened peritoneum, with encapsulation of the liver, transverse colon, and stomach.’ A final diagnosis of EPS was made based on the operative findings. She underwent right hemicolectomy with loop-end ileostomy, after which she had waxing and waning abdominal pain. Continuous renal replacement therapy was used in lieu of intermittent hemodialysis; PD was not resumed. Ultimately, the patient’s overall condition failed to improve; she remained in the ICU for several weeks due to continued vasopressor requirements before ultimately expiring from cardiac arrest.

## Discussion

EPS is a rare complication of PD, often resulting from the chronic use of high-glucose concentrations in the dialysate to manage volume overload. Heat sterilization of the dialysate forms glucose degradation products that promote the formation of advanced glycation end-products. These accumulate in the peritoneal mesothelium, stimulating peritoneal macrophages and ultimately resulting in sclerosis [2]. The symptomatic presentation of EPS is similar to bowel obstruction, with nausea, vomiting, secondary cachexia, and weight loss. Notably, the diagnosis of EPS in 70-90% of cases occurred after the withdrawal of PD and transition to conventional hemodialysis. A median time of five years on PD before the occurrence of EPS has been suggested. It is also theorized that EPS may begin developing before PD cessation but becomes more clinically severe afterwards [3].

The case above highlights several known risk factors for the development of EPS, including clinical features of obstruction, history of prolonged PD, and worsening condition after the cessation of PD. As such, it has instructive value for clinicians to consider EPS when patients on PD present with abdominal pain and obstruction that does not improve with an aggressive bowel regimen. EPS has a morbidity of 50% within one year of diagnosis. Once EPS is identified, corticosteroids are the drug of choice due to suppressing inflammation and preventing fibrin deposition. Combination therapy with tamoxifen (a selective estrogen receptor modulator) has also been shown to decrease mortality through inhibition and modulation of transforming growth factor beta (TGF-β) [3,4]. Surgical enterolysis of the thickened peritoneum has also been associated with improved outcomes, with one retrospective study of 50 patients showing a 96% success rate [5]. However, surgical intervention is generally reserved for patients with active obstruction due to the significant risks associated with performing surgery in chronic cases (e.g., adhesions, perforations) [6].

This case not only serves as a lesson in the management of refractory abdominal pain in patients on PD but also highlights the underreported role of SLE as a significant risk factor for developing EPS. A retrospective cohort study of 26 SLE patients undergoing PD found that two patients (8%) developed EPS, both of whom were on long-term PD (6-10 years) [7]. Comparatively, a separate study analyzing the general incidence of EPS in relation to the duration of PD found incidence rates of 0.3%, 0.8%, and 3.9% at three, five, and eight years on PD, respectively [8]. These findings suggest that SLE may increase the relative risk of developing EPS, in some cases doubling the risk.

SLE is known to cause inflammation of the peritoneal serosa through immune complex deposition, which triggers the destruction of the mesothelium, stimulation of macrophages, and subsequent production of cytokines such as TGF-β, which accelerate fibrosis [9]. In patients with SLE undergoing PD with high-glucose dialysates, this dual insult to the peritoneum may potentially lead to increased immune-mediated fibrosis and sclerosis [7,8]. There is no definitive strategy to prevent the development of EPS; however, increased time spent on PD is a definite risk factor. While no studies have determined the optimal duration of PD, researchers in Japan have suggested transitioning to hemodialysis after eight years [10]. Other theorized preventative strategies include peritoneal lavage and the utilization of more biocompatible dialysates [4,11]. Further studies are needed to develop an evidence-based approach for the prevention of EPS, especially in high-risk populations.

## Conclusions

EPS is a complication of chronic PD with a poor prognosis; it may result in bowel obstruction due to extensive accumulation of fibrocollagenous material in the peritoneal cavity. Patients with SLE are at greater risk of developing EPS due to the increased inflammatory burden on the peritoneal serosa from immune complex deposition. Hence, clinicians should maintain a high index of suspicion for EPS in chronic PD patients with comorbid SLE presenting with abdominal pain and constipation. Prompt recognition of EPS and timely intervention can help minimize disease progression and improve clinical outcomes. Preventive measures such as limited duration of PD and the use of more biocompatible PD fluid should be further studied to help reduce the incidence of EPS in this high-risk population.
